# Factors Influencing Left Ventricular Thrombus Recurrence After Resolution

**DOI:** 10.3390/jcdd13090412

**Published:** 2026-08-25

**Authors:** Lili Xu, Lixiang Deng, Zhenzhen Huang, Kuan Cheng, Ye Xu, Yunlong Ling, Guijian Liu, Chaofeng Chen, Tao Yu, Quan Li, Wenqing Zhu, Yang Pang, Qingxing Chen, Junbo Ge

**Affiliations:** 1 Department of Cardiology, Shanghai Institute of Cardiovascular Diseases, Zhongshan Hospital, Fudan University, National Clinical Research Center for Interventional Medicine, Shanghai 200032, Chinazhu.wenqing@zs-hospital.sh.cn (W.Z.); jbge@zs-hospital.sh.cn (J.G.); 2 Department of Cardiology, Shanghai Geriatric Medical Center, Shanghai 201104, China; 3 The Second People’s Hospital of Kashgar Prefecture, Kashgar 844000, China

**Keywords:** left ventricular thrombus, recurrence, risk factors

## Abstract

**Background**: Left ventricular thrombus (LVT) is a serious complication associated with cardiomyopathy and impaired left ventricular (LV) systolic function. Patients with a resolved LVT remain at risk for recurrence and subsequent thromboembolism. However, the factors influencing the recurrence of LVT are not yet fully understood. The aim of this study was to identify the risk factors and clinical outcomes related to LVT recurrence and to improve follow-up strategies and treatment options. **Methods and results**: We retrospectively investigated patients confirmed to have a resolved LVT by transthoracic echocardiography from January 2018 to April 2021 at Zhongshan Hospital Fudan University. All patients received anticoagulant therapy for more than 6 months and underwent at least two follow-up transthoracic echocardiograms. No statistically significant differences were observed in baseline characteristics between the LVT recurrence and non-recurrence groups, including gender, age, diabetes, hyperlipidemia, renal function, previous stroke history, other underlying medical conditions, ejection fraction, or left ventricular diameter. Patients in the recurrence group exhibited a higher prevalence of previous myocardial infarction and percutaneous coronary intervention compared to the non-recurrence group (84.6% vs. 61.1%, *p* = 0.03; 76.9% vs. 52.8%, *p* = 0.03). Additionally, patients in the recurrence group tended to have more ventricular aneurysms (50.0% vs. 22.2%, *p* = 0.008) and larger previous thrombus sizes (27.7 ± 12.6 vs. 21.4 ± 9.1 mm, *p* = 0.008) compared to those in the non-recurrence group. Multivariate logistic regression analysis indicated that the longitudinal diameter of the LVT was an independent risk factor for LVT recurrence (OR 1.058, 95% CI 1.003–1.115, *p* = 0.04). ROC curve analysis revealed an area under the curve of 0.647 for the longitudinal diameter of the LVT, with an optimal cut-off value of 23.5 mm, a sensitivity of 62%, and a specificity of 64%. After a follow-up duration of 3.0 ± 2.5 years, the incidence of non-fatal myocardial infarction and major adverse cardiovascular events (MACEs) in the recurrence group was significantly higher than that in the non-recurrence group [non-fatal myocardial infarction: 3 (11.5%) vs. 1 (1.4%), *p* = 0.02; MACE: 7 (26.9%) vs. 7 (9.7%), *p* = 0.01]. No statistically significant differences were found in bleeding events, systemic embolism, or all-cause death between the two groups. **Conclusions**: Our study indicates that a history of myocardial infarction, the presence of ventricular aneurysm, and a larger thrombus diameter are significant factors influencing LVT recurrence. Furthermore, the longitudinal diameter of the thrombus is identified as an independent risk factor for recurrence following resolution. The threshold for LVT diameter requiring extended anticoagulation and the required duration of anticoagulation need to be confirmed in future studies.

## 1. Introduction

Left ventricular thrombus (LVT) is a common complication in patients with heart failure due to ischemic or non-ischemic cardiomyopathy [1]. Left ventricular dysfunction has been identified as a significant independent predictor of LVT [2]. Moreover, LVT is closely associated with a high risk of severe systemic thromboembolism, as well as increased morbidity and mortality [3,4]. Currently, anticoagulation therapy remains the primary strategy in the management of LVT [5], yet some patients experience recurrence of LVT after initial resolution, presenting a great challenge in clinical practice [6,7]. However, the factors influencing LVT recurrence remain unclear.

The current guidelines recommend 3 to 6 months of anticoagulation therapy for LVT [8,9]. Studies have shown that complete resolution of LVT occurs in approximately 62.3% of patients with smaller thrombi within 3 months [10]. Although the majority of patients achieve LVT resolution after appropriate anticoagulation therapy, some patients may still experience recurrence following discontinuation of treatment [11,12]. Increasing the dose of oral anticoagulants to prevent recurrence may lead to a higher bleeding risk, which is not an optimal strategy for managing LVT recurrence [13,14]. However, there are limited data concerning recurrence of thrombus following resolution and the potential clinical outcomes [15]. In this study, we aimed to identify the risk factors for and clinical outcomes associated with LVT recurrence after resolution and to inform follow-up strategies and treatment approaches in clinical practice.

## 2. Methods

### 2.1. Study Design

This was a single-center, retrospective study conducted at Zhongshan Hospital, Fudan University. We included a total of 98 patients who were confirmed to have a resolved LVT from 1 January 2018 to 1 January 2021 and had received anticoagulation therapy for 6 months or more. The exclusion criteria included (1) LVT caused by non-cardiovascular diseases, such as autoimmune disease and malignant tumors, (2) patients in the end stages of various diseases, and (3) patients lost to follow-up after LVT resolution. Patients were categorized into the recurrence and non-recurrence groups based on transthoracic echocardiography (TTE) findings. LVT recurrence was defined as new detection of LVT on follow-up TTE after prior complete resolution of the initial LVT. The longitudinal diameter of the LVT was measured after discontinuation of anticoagulant therapy. This study was approved by the Ethics Committee of Zhongshan Hospital, Fudan University, and complied with the Declaration of Helsinki.

### 2.2. Demographic and Clinical Characteristics

Baseline characteristics collected included age, gender, history of percutaneous coronary intervention (PCI), and history of coronary artery bypass grafting (CABG), as well as comorbidities such as hypertension, diabetes, hyperlipidemia, atrial fibrillation (both paroxysmal and persistent), cardiomyopathy, heart failure, stroke, previous myocardial infarction, and chronic kidney disease. All patients underwent echocardiographic evaluations, recording left atrial diameter, left ventricular end-diastolic diameter, left ventricular end-systolic diameter, left ventricular ejection fraction (LVEF), LVT area (calculated as longitudinal diameter × transverse diameter), and the presence of ventricular aneurysm.

The presence of LVT was assessed using TTE and defined as hypoechoic masses located near myocardial segments, with reduced or impaired left ventricular motion. LVTs must have been visible on at least two different views throughout the entire cardiac cycle. The location, size, and mobility of the LVTs were recorded. Echocardiography was repeated 3 months after the initial detection of a ventricular thrombus, and the size of the LVT was evaluated based on the thrombus area obtained from the imaging examination.

Thrombus resolution was defined as the complete disappearance of LVT at follow-up. Ineffective anticoagulation was characterized by an unchanged or increased LVT area. Major adverse cardiovascular events, including ischemic stroke, acute myocardial infarction or acute systemic arterial embolism, major bleeding events, and all-cause mortality, were also recorded.

### 2.3. Follow-Up

The follow-up duration was defined as the period of time that elapsed until confirmation of LVT recurrence or non-recurrence, as assessed during the last follow-up echocardiogram after LVT resolution. The median follow-up time was 3.0 years. The enrolled patients completed two or more echocardiographic follow-ups at our hospital.

### 2.4. Statistical Analysis

Continuous variables were expressed as mean ± standard deviation. Normally distributed variables were compared using the t-test, while non-normally distributed variables were analyzed using the rank-sum test. Categorical variables were presented as percentages and compared using chi-square test or Fisher’s exact test. Multivariate logistic regression was utilized to identify independent predictors of ineffective anticoagulation treatment for LVT. ROC curves were generated to determine the optimal critical value, sensitivity, and specificity. A *p* value less than 0.05 was considered statistically significant.

## 3. Results

### 3.1. Demographic and Clinical Characteristics

A total of 98 patients with LVT resolution were enrolled in this study. They were classified into two groups based on LVT recurrence, including 26 patients (26.5%) in the recurrence group and 72 patients (73.5%) in the non-recurrence group. The patients in the two groups were similar in terms of average age, gender, and previous history of hypertension, diabetes, hyperlipidemia, atrial fibrillation, chronic kidney disease, and stroke. The recurrence group showed a higher prevalence of prior myocardial infarction (recurrence vs. non-recurrence: 84.6% vs. 61.1%, *p* = 0.03) and PCI (recurrence vs. non-recurrence: 76.9% vs. 52.8%, *p* = 0.03). Conversely, patients in the non-recurrence group were more likely to suffer non-ischemic cardiomyopathy (recurrence vs. non-recurrence: 11.5% vs. 38.9%, *p* = 0.01). Based on echocardiographic examination, patients in the recurrence group were more likely to have a ventricular aneurysm compared to the non-recurrence group (50.0% vs. 22.2%, *p* = 0.008). Additionally, patients in the recurrence group had a greater LVT longitudinal diameter (27.7 ± 12.6 mm vs. 21.4 ± 9.1 mm, *p* = 0.008). There were no significant differences between the two groups regarding the use of warfarin, rivaroxaban, and dabigatran. However, the administration of aspirin and clopidogrel was notably higher in the recurrence group (aspirin: 65.4% vs. 40.3%, *p* = 0.03; clopidogrel: 69.2% vs. 47.2%, *p* = 0.05) (Table 1).

### 3.2. Multivariate Analysis of Predictors for LVT Recurrence

Logistic regression analysis, considering factors such as previous PCI, myocardial infarction, ventricular aneurysm, and LVT longitudinal diameter, revealed that the LVT longitudinal diameter is an independent risk factor for LVT recurrence (OR 1.058, 95% CI 1.003–1.115, *p* = 0.04) (Table 2).

### 3.3. Value of LVT Longitudinal Diameter in the Prediction of LVT Recurrence

**Figure 1:**
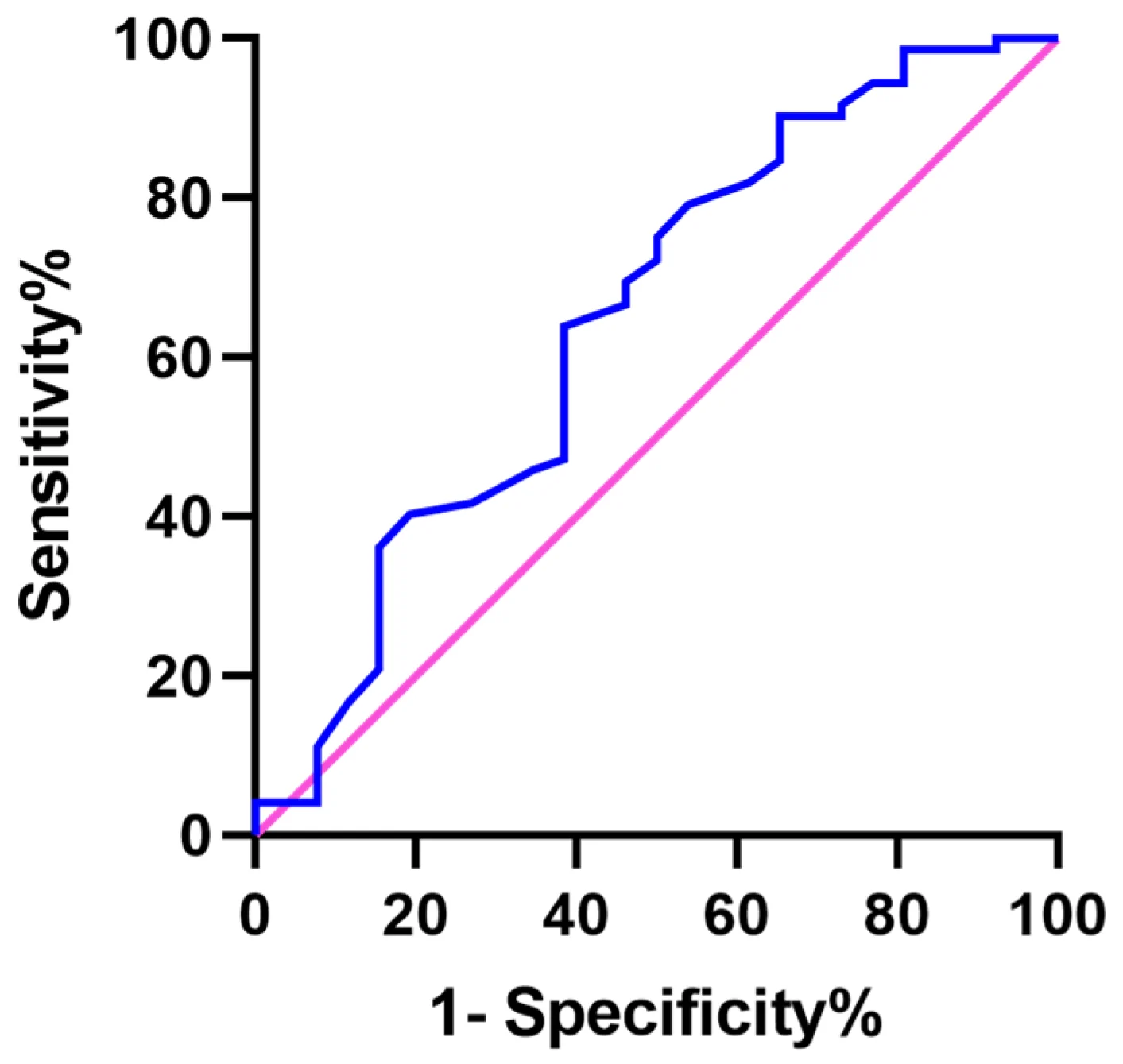
ROC curve of thrombus longitudinal diameter for predicting thrombus recurrence.

Using a ROC curve, the optimal cut-off value for LVT longitudinal diameter was determined through the maximum Youden index. The results indicated that the area under the curve (AUC) for LVT longitudinal diameter was 0.647, with an optimal cut-off value of 23.5 mm, a sensitivity of 62%, and a specificity of 64% (Figure 1).

### 3.4. Follow-Up

After 3.0 ± 2.5 years of follow-up, no statistical difference in major bleeding, ischemic stroke, or all-cause mortality was observed between the two groups. However, the incidence of non-fatal myocardial infarction and major adverse cardiovascular events (MACEs) was significantly higher in the recurrence group compared to the non-recurrence group [(myocardial infarction: 3 (11.5%) vs. 1 (1.4%), *p* = 0.02); MACE: 7 (26.9%) vs. 7 (9.7%), *p* = 0.01] (Table 3).

## 4. Discussion

LVT is a common complication associated with cardiomyopathy and heart failure, leading to adverse cardiovascular events such as stroke and systemic embolism, which contribute to high mortality and disability. Hypercoagulable state, circulatory stasis, and myocardial injury, also known as Virchow’s triad, are the basic components of LVT formation [16]. A prethrombotic state can be reversed by the application of anticoagulants. Nevertheless, the risk of recurrence persists once the medication is discontinued, due to ongoing risk factors for thrombosis. Although previous studies have demonstrated a high rate of LVT resolution with anticoagulation treatment, there are limited reports on LVT recurrence following resolution [17,18,19]. Additionally, best practice for the long-term management of these patients remains unclear [20]. Therefore, it is crucial to identify risk factors for LVT recurrence after resolution to guide management of high-risk patients by implementing more frequent follow-up and longer anticoagulation treatment. Our study showed that the percentages of patients with previous myocardial infarction, history of PCI, and the presence of ventricular aneurysm were significantly higher, and the longitudinal diameters of the thrombi were notably larger, in the recurrence group compared with the non-recurrence group.

LVT formation is primarily associated with abnormal ventricular wall motion, hemodynamic changes, and a hypercoagulable state induced by cardiac conditions such as myocardial infarction, cardiomyopathy, and valvular heart disease [21]. Continuous anticoagulation treatment is essential to prevent LVT recurrence [22,23]. Nonetheless, it cannot eliminate the underlying risk factors for thrombosis formation. Zhou XD et al. found that nearly 25% of patients with LVT resolution experience recurrence within 1 year, and that LV aneurysm is a significant factor associated with recurrence [15]. When risk factors persist, LVTs tend to recur. In addition, inconsistent medication use and poor patient compliance with anticoagulation therapy may also contribute to recurrence following resolution of a thrombus.

Anticoagulation therapy is the primary treatment regimen for LVT. The duration of anticoagulation treatment directly influences thrombus resolution and the risk of recurrence. A short anticoagulation treatment span is an independent risk factor for LVT recurrence after resolution [7,23,24,25]. Prolonged anticoagulation has been shown to reduce the risk of recurrence, particularly in patients with a larger thrombus diameter [11,12]. Larger thrombi can significantly alter blood flow dynamics in the left ventricle, leading to decreased local blood flow velocity and vortex formation, which increases the risk of thrombus recurrence [10,26]. Previous studies have indicated that patients with a larger thrombus area are at a higher risk of adverse events such as systemic embolism [27,28]. In a retrospective study involving 159 patients with confirmed LVT recurrence, the presence of LVT was associated with an extremely high risk of MACEs (n = 59, 37.1%) and embolic complications (n = 35, 22.2%). The study also suggested that anticoagulation treatment lasting more than 3 months is independently associated with lower rates of MACEs. The authors emphasized the importance of identifying predictors of LVT recurrence following resolution. However, the risk of bleeding caused by intensified anticoagulation remains a concern; therefore, a comprehensive risk–benefit assessment becomes urgent. The study also revealed that thrombus length was an independent risk factor for LVT recurrence after resolution [10].

For LVTs longer than 23.5 mm, it is recommended to extend anticoagulation time; this finding represents a novel contribution of our study. Prolonging anticoagulant treatment for patients at high risk of thrombus recurrence can theoretically help maintain hypocoagulability of the blood and reduce the risk of thromboembolism. Based on the prior literature and our single-center experience, we proposed lifelong anticoagulation with direct oral anticoagulant (DOAC). However, these findings need to be confirmed by prospective studies with larger sample sizes.

## 5. Limitations

This was a single-center retrospective study exploring factors influencing LVT recurrence after resolution. The sample size was relatively small. Moreover, the two-dimensional planar measurements of a thrombus may not fully represent its three-dimensional structure. In future studies, we will collect relevant case data, expand the sample size, incorporate advanced imaging modalities, and design prospective multi-center randomized controlled studies. We will also arrange for regular follow-ups to further assess the efficacy and safety of longer anticoagulation durations in patients with LVT.

## 6. Conclusions

In the present study, we found that previous myocardial infarction, ventricular aneurysm, and larger thrombus diameter are significant factors placing patients at high risk for LVT recurrence. Notably, the longitudinal diameter of the thrombus is an independent risk factor for recurrence following LVT resolution; however, the threshold for LVT diameter requiring extended anticoagulation and the required duration of anticoagulation need to be confirmed in future studies.

Our study provides a comprehensive summary of the disease characteristics of patients with LVT and analyzes LVT longitudinal diameter. These findings can offer a reliable basis for further interventional clinical trials and serve as a reference for determining appropriate anticoagulation time in clinical practice, ultimately delivering important therapeutic benefits for patients with LVT.

## Figures and Tables

**Table 1:** Baseline characteristics of patients in the recurrence and non-recurrence groups.

	Recurrence (N = 26)	Non-Recurrence (N = 72)	*p* Value
Male sex, n (%)	25 (96.2%)	65 (90.3%)	0.35
Age, years	57.8 ± 13.3	53.3 ± 14.5	0.17
Medical history			
Hypertension, n (%)	10 (38.5%)	27 (37.5%)	0.93
Diabetes mellitus, n (%)	6 (25.5%)	16 (28.9%)	0.93
Hyperlipidemia, n (%)	3 (11.5%)	1 (1.4%)	0.10
Atrial fibrillation, n (%)	3 (11.5%)	9 (12.5%)	0.90
Chronic kidney disease			
eGFR (mL/min/1.73 m^2^, $\bar{x}$ ± s)	78.4 ± 21.2	77.3 ± 19.8	0.82
Previous stroke, n (%)	2 (7.7%)	2 (2.8%)	0.61
Myocardial infarction, n (%)	22 (84.6%)	44 (61.1%)	0.03
PCI, n (%)	20 (76.9%)	38 (52.8%)	0.03
CABG, n (%)	3 (11.5%)	7 (9.7%)	0.79
Non-ischemic cardiomyopathy, n (%)	4 (11.5%)	28 (38.9%)	0.01
Dilated cardiomyopathy, n (%)	2 (3.8%)	14 (19.4%)	0.06
Hypertrophic cardiomyopathy, n (%)	2 (7.7%)	3 (4.2%)	0.48
Post-myocarditis cardiomyopathy, n (%)	0 (0%)	1 (1.4%)	-
Echocardiogram parameters			
LVEF < 50%, n (%)	22 (84.6%)	66 (91.7%)	0.31
LVEF (%)	43.5 ± 10.0	38.6 ± 11.6	0.06
Aneurysm, n (%)	13 (50.0%)	16 (22.2%)	0.008
LVEDD (mm)	55.0 ± 6.4	56.5 ± 8.4	0.42
LVESD (mm)	39.4 ± 8.1	43.4 ± 10.7	0.09
LAD (mm)	42.7 ± 6.4	42.9 ± 6.9	0.85
Mitral regurgitation, n (%)	1 (3.8%)	4 (5.6%)	0.73
LVT area (mm^2^)	368.9 ± 220.4	310.3 ± 207.9	0.23
LVT longitudinal diameter (mm)	27.7 ± 12.6	21.4 ± 9.1	0.008
LVT transverse diameter (mm)	12.9 ± 3.9	13.3 ± 5.2	0.71
Medication use, n (%)			
Warfarin	22 (84.6%)	53 (73.6%)	0.26
Rivaroxaban	3 (11.5%)	13 (18.1%)	0.44
Dabigatran	1 (3.9%)	6 (8.3%)	0.68
Aspirin	17 (65.4%)	29 (40.3%)	0.03
Clopidogrel	18 (69.2%)	34 (47.2%)	0.05
Metoprolol	24 (92.3%)	65 (90.3%)	0.76
ACEI/ARB	20 (76.9%)	59 (81.9%)	0.58

PCI, percutaneous coronary intervention; CABG, coronary artery bypass grafting; LVEF, left ventricular ejection fraction; LVEDD, left ventricular end-diastolic diameter; LVESD, left ventricular end-systolic diameter; LAD, left atrial diameter; LVT, left ventricular thrombus; ACEI, angiotensin-converting enzyme inhibitor; ARB, angiotensin receptor blocker.

**Table 2:** Logistic regression analysis of independent predictors of LVT recurrence.

	OR (95% CI)	*p* Value
PCI	4.145 (0.903–19.022)	0.07
Myocardial infarction	0.764 (0.134–4.346)	0.76
Aneurysm	2.896 (0.931–9.008)	0.07
LVT longitudinal diameter (mm)	1.058 (1.003–1.115)	0.04

OR, odds ratio; PCI, percutaneous coronary intervention; LVT, left ventricular thrombus.

**Table 3:** Adverse events in the recurrence and non-recurrence groups after LVT resolution.

	Recurrence (N = 26)	Non-Recurrence (N = 72)	*p* Value
Major bleeding events	1 (3.8%)	2 (2.8%)	0.79
Stroke/transient ischemic attack	2 (7.7%)	3 (4.2%)	0.70
Myocardial infarction	3 (11.5%)	1 (1.4%)	0.02
All-cause death	2 (7.7%)	1 (1.4%)	0.11
Major adverse cardiovascular events	7 (26.9%)	7 (9.7%)	0.01

## Data Availability

The datasets generated or analyzed during the current study are available from the corresponding author upon reasonable request.
